# Bovine serum albumin further enhances the effects of organic solvents on increased yield of polymerase chain reaction of GC-rich templates

**DOI:** 10.1186/1756-0500-5-257

**Published:** 2012-05-24

**Authors:** Eric M Farell, Gladys Alexandre

**Affiliations:** 1 Department of Biochemistry, Cellular and Molecular Biology, University of Tennessee, Knoxville, TN 37996, USA

## Abstract

**Background:**

While being a standard powerful molecular biology technique, applications of the PCR to the amplification of high GC-rich DNA samples still present challenges which include limited yield and poor specificity of the reaction. Organic solvents, including DMSO and formamide, have been often employed as additives to increase the efficiency of amplification of high GC content (GC > 60%) DNA sequences. Bovine serum albumin (BSA) has been used as an additive in several applications, including restriction enzyme digestions as well as in PCR amplification of templates from environmental samples that contain potential inhibitors such as phenolic compounds.

**Findings:**

Significant increase in PCR amplification yields of GC-rich DNA targets ranging in sizes from 0.4 kb to 7.1 kb were achieved by using BSA as a co-additive along with DMSO and formamide. Notably, enhancing effects of BSA occurs in the initial PCR cycles with BSA additions having no detrimental impact on PCR yield or specificity. When a PCR was set up such that the cycling parameters paused after every ten cycles to allow for supplementation of BSA, combining BSA and organic solvent produced significantly higher yields relative to conditions using the solvent alone. The co-enhancing effects of BSA in presence of organic solvents were also obtained in other PCR applications, including site-directed mutagenesis and overlap extension PCR.

**Conclusions:**

BSA significantly enhances PCR amplification yield when used in combination with organic solvents, DMSO or formamide. BSA enhancing effects were obtained in several PCR applications, with DNA templates of high GC content and spanning a broad size range. When added to the reaction buffer, promoting effects of BSA were seen in the first cycles of the PCR, regardless of the size of the DNA to amplify. The strategy outlined here provides a cost-effective alternative for increasing the efficiency of PCR amplification of GC-rich DNA targets over a broad size range.

## Background

Ever since the introduction of the Polymerase Chain Reaction [1], it has been one of the most often used tools in molecular biology, and has played a role in many of the major advances in Biology including cloning [2], mutagenesis [3], even with small amounts of DNA target [4]. This technique is not without its limitations though, as some DNA templates have proved difficult to amplify. The most common reason for troublesome amplification lies in target DNA sequences that have high GC content (GC content >60%) [5]. Many studies have been undertaken to identify experimental modifications that would alleviate or eliminate this problem altogether, with most studies focusing mainly on primer design [5-7], altering the cycling parameters [8,9], and the use of PCR additives [10-17]. PCR additives most often employed are organic co-solvents such as DMSO and formamide [12,13,15,16]. DMSO has been found to significantly increase the yield of a PCR reaction on GC-rich DNA templates, by preventing the formation of secondary structures [5]. The effects of formamide are less clear and still debated, with some studies indicating that formamide greatly increases specificity of amplification of GC-rich DNA templates and others failing to detect any effect [14,16]. Formamide also appears to be effective only within a narrow concentration range [10] which may be related to the fact that formamide is postulated to bind to the grooves in DNA, thus destabilizing the double helix and perhaps improving initial melting [10]. Bovine serum albumin (BSA) has been applied to many laboratory molecular techniques, including restriction enzyme digestions of DNA to increase the thermal stability and half-life of the restriction enzymes in the reactions [18]. For this reason, its effects have also been investigated in PCR and several studies have demonstrated that BSA have a beneficial effect on the yield of PCR (and qPCR) amplification of ancient DNA or of DNA found in extracts from feces, freshwater, or marine water [19,20]. The beneficial effects of BSA were observed in the absence of any other additive. Since, most of the PCR inhibitors in the samples analyzed in these experiments were also substances that BSA can bind to, the beneficial effects of BSA were proposed to prevent these inhibitors from interacting with DNA (Taq) polymerase [19]. When used in PCR amplification from genomic DNA that is free of any PCR inhibitors, BSA has not been shown to have a significant effect on specificity or amplification yield [13]. In fact, the effect of BSA on PCR has not been systematically analyzed. Here, we use BSA in conjunction with organic solvents, DMSO or formamide, to amplify NA templates of high GC content. Our results demonstrate that when used with organic solvents, BSA acts as a powerful co-enhancer of PCR amplification of these DNA templates. We also provide evidence that supports the notion that one of the reasons that its effects have gone unnoticed is due to the fact that BSA is sensitive to high temperatures of PCR, and rapidly loses its enhancing abilities. Adding BSA to PCR reactions in presence of organic solvents also allows high PCR yields of GC-rich DNA of various sizes to be obtained while reducing the concentration of solvent used. Using the genomic DNA of the alphaproteobacterium *Azospirillum brasilense* Sp7 [20] which has a GC content above 65% [21], we have tested various cycling parameters and combination of additives to amplify DNA fragments ranging from 392 to 7,103 base pairs, (with each having a GC content of 66% or greater). For this study, the DNA sequences corresponding to regions of interest were retrieved from the draft genome sequence of *Azospirillum brasilense* (http://genome.ornl.gov/microbial/abra/19sep08/) and from sequences available in the NCBI GenBank database. The DNA templates were a 392 base pair fragment with a GC content of 66% (che1P) (NCBI GI 17864024), a 798 base pair fragment with a GC content of 68% (tlp5) (contig 115, or2365), a 1,641 base pair fragment with a GC content of 73% (cheA4) (contig 120, or3019), a 2,638 base pair fragment with a GC content of 66% (tlp2) (contig 213, or4271), a 3,389 base pair fragment with a GC content of 68% (cheA1) (NCBIGI17864025), and a 7,103 base pair fragment with a GC content of 68% (cheOp1) (NCBI GI 17864024). We also applied this protocol in “Touchdown” PCR, as well as in an overlap extension PCR and in combination with a widely used commercialized site-directed mutagenesis kit (Stratagene Quickchange Site Directed Mutagenesis Kit, Stratagene) with primers designed to introduce a single amino acid change. Our results highlight a strategy and experimental conditions for using BSA as a co-enhancer that significantly increases PCR yields when used with solvent additives in various PCR applications.

## Findings

**Figure 1:**
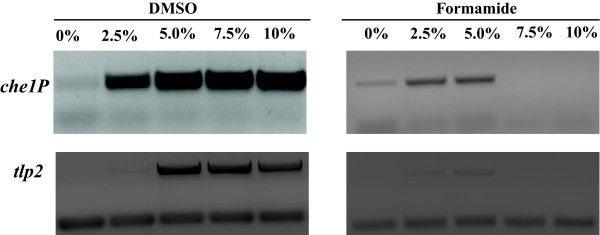
**Effects of DMSO and formamide on the PCR amplification of GC-rich templates.** Typical examples of the effects of DMSO and formamide addition at different concentrations on the PCR amplification of 2–3 kb GC-rich DNA templates. **A)** Effect of DMSO on PCR amplification of che1P (top) and tlp2 (bottom) as observed by gel electrophoresis of total PCR products. The gels were stained with ethidium bromide and photographed. **B)** Effect of formamide on PCR amplification of che1P (top) and tlp2 (bottom) in comparable experiments.

**Figure 2:**
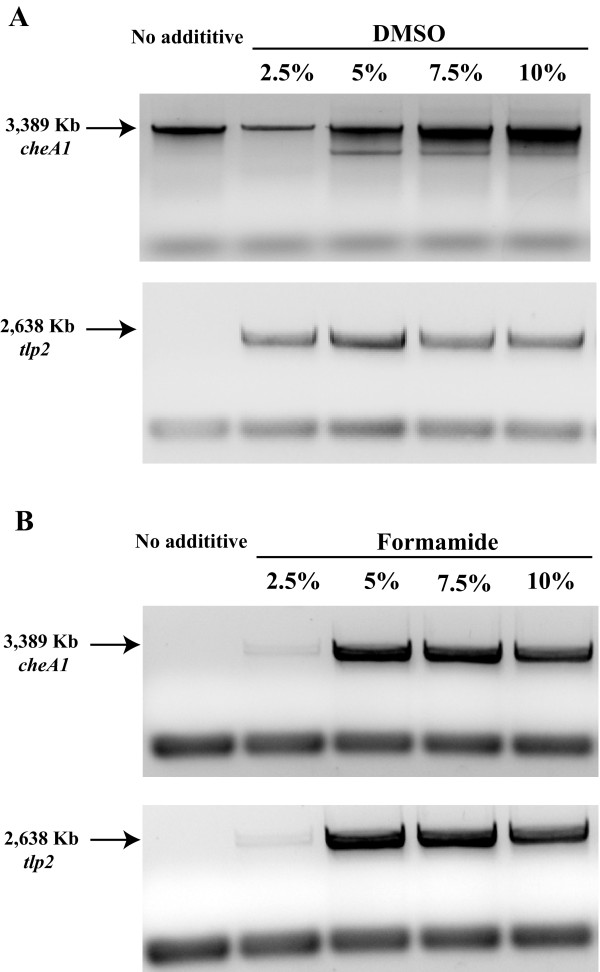
**PCR yield enhancing effect of BSA as used as a co-additive with organic solvents.** Typical examples of the effects of BSA as a co-additive with DMSO and formamide, at different concentrations, on PCR amplification of GC-rich DNA templates. **A)** Effect of DMSO on PCR of che1P (left) and combined effect of DMSO with 6 μg/μl BSA (right) as observed by gel electrophoresis of total PCR product. **B)** Effects of formamide on PCR amplification of che1P (left) and combined effect of formamide with 6 μg/μl BSA (right) in comparable experiments.

**Figure 3:**
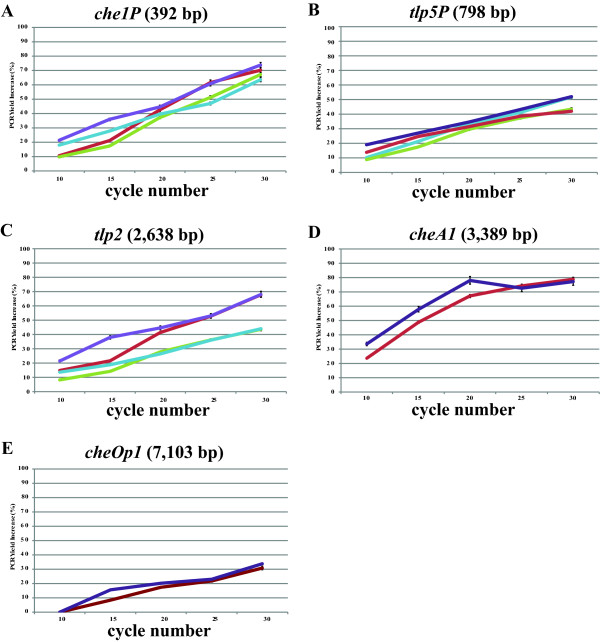
**Effect of BSA addition as a co-enhancer with organic solvents on PCR Yield.** The PCR yield increase is expressed relative to the PCR yield obtained without any additive. On all panels, the following legend applies: red line, DMSO (7.5%) only; green line, formamide (2.5%) only; purple line, DMSO (7.5%) and BSA (6 μg/μl); light blue, formamide (2.5%) and BSA (6 μg/μl). **A)** PCR amplification of che1P (392 bp); **B)** PCR amplification of tlp5P (798 bp); **C)** PCR amplification of tlp2 (2,638 bp); **D)** PCR amplification of cheA1 (3,389 bp); **E)** PCR amplification of cheOP1 (7,103 bp).

**Figure 4:**
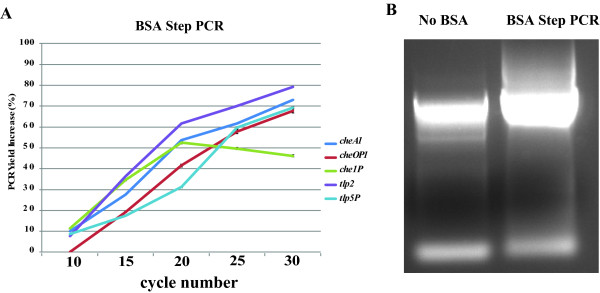
**Effect of BSA as a co-additive with organic solvents when supplemented at intermediate steps of PCR. A)** Effect of BSA addition on the PCR yield when added every ten cycles for DNA fragments of broad size range (392 bp to 7,103 bp); **B)** Representative example of the effect of BSA addition on the PCR amplification of cheA1 (3,389 bp) in the presence of DMSO, as detected by analysis of DNA fragments amplified by gel electrophoresis.

**Figure 5:**
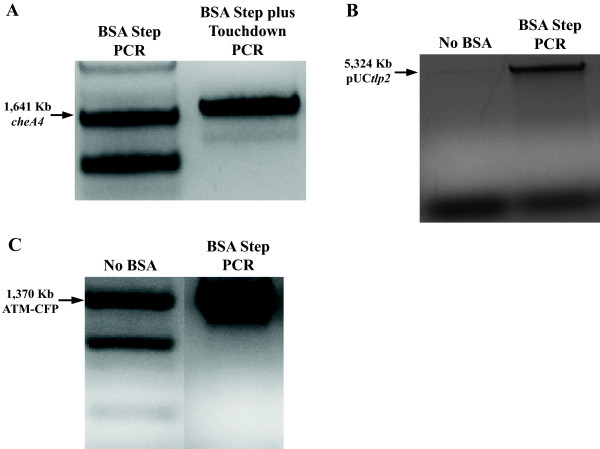
**Effect of BSA as a co-additive with organic solvents in various PCR applications. A)** A representative example of the effect of BSA addition (BSA Step) as a co-enhancer of DMSO effect on PCR amplification of cheA4 in a “Touchdown” PCR protocol, as detected by gel electrophoresis. **B)** A representative example of the effect of BSA Step protocol used in site-directed mutagenesis (QuickChange site-directed mutagenesis, Stratagene) PCR amplification of pUCtlp2, as detected by gel Electrophoresis. **C)** A representative example of the effect of BSA Step protocol used in an overlap extension PCR application, fusing ATM with CFP to yield ATM-CFP, as detected by gel Electrophoresis.

Of the DNA fragments that we initially attempted to amplify, only tlp2, che1P, and cheA1 (Table 1) produced a PCR amplification product without the use of solvent additives, when genomic DNA of *A. brasilense* was used a s a template (Figure 1). The effects of DMSO at 1.25%, 2.5%, 5.0%, 7.5%, and 10%, (w/vol) as well as formamide at similar concentrations as additives in PCR were first tested (Figure 1). A set of PCR was also run with BSA at 1–10 μg/μl (data not shown). Consistent with data from the literature [13], while both DMSO or formamide addition in PCR promoted increased yield of amplification of DNA fragments of 2–3 kb, the enhancing effect of DMSO was greater than that of formamide (Figure 1). Under these conditions, we also observed that increasing the concentration of DMSO could also lead to decrease in PCR specificity (Figure 2). On the other hand, the effect of formamide in promoting increased PCR yield decreased for DNA fragments larger than about 2.5 kb (Figure 1). There was no significant effect of adding BSA alone as a PCR additive, under these conditions, including no detrimental effect (Data not shown). The enhancing effects of BSA on PCR yields were detected when used in combination with DMSO or formamide, over a broad range of DNA fragment sizes (Figure 2). Furthermore, BSA addition broadened the range of concentrations for which the organic solvent could be added, even for DNA templates of larger sizes (Figure 2). The consensus of the current literature on formamide indicates that it is most effective as a PCR additive when used at concentrations ranging from 0 to 5%, with effectiveness dropping off completely at 10% [10]. Our results indicate that in presence of BSA, formamide is effective at least up to concentrations of 10% and with DNA templates up to 2.5 kb in size (tlp2); however, it failed to promote amplification of DNA fragments of larger sizes (Figure 1). This result is consistent with the reported effect of formamide as being most efficient for DNA template amplifications up to about 2.5 kb [10,15] and further support the notion that DMSO and formamide enhanced PCR yields by different mechanisms(s). Regardless of these differences, addition of BSA to this PCR appeared to further promote and expand the beneficial effects seen when either of the two solvents was used as a PCR additive. Given the potential negative effects that high organic solvent concentrations may have on sensitive downstream applications such as sequencing or cloning, the enhancing effect of BSA addition is significant. In order to gain insight into how BSA might act as a co-enhancer with DMSO or formamide, we analyze the effect of its addition on amplification yield over 10, 15, 20, 25, and 30 cycles of PCR. This analysis confirmed that adding DMSO or formamide, but not BSA alone, to PCR leads to increase in yield (Figure 3). When BSA was used as a co-enhancer with DMSO or formamide, an increase in yield could be detected in the first 15 cycles only for all templates (Figure 3). This increase ranged from a 10.5% (che1P) increase in yield to a 22.7% increase of yield (cheA1) throughout the course of the first 15 cycles. Furthermore, the effective concentration of BSA was found to increase with the size of the DNA fragment amplified up to a maximum BSA concentration (10 μg/μl) where no further increase in yield was detected. In addition, no decrease in yield was observed even with the greatest concentration of BSA added under these conditions (Figure 4). The cycle-limited enhancing effect of BSA on the PCR yield suggested that this protein may become denatured over time, thereby losing its effectiveness. To test this hypothesis a PCR was set up in which DMSO or formamide was combined with BSA in the initial reaction buffer at the most effective concentrations determined above, and ran for 10 cycles before addition of fresh solution of BSA (0–10 μg/μl final concentration). BSA addition was repeated over 30 PCR cycles and the effect on yield was analyzed as described above. A sustained increase in yield could be detected when BSA was added at every tenth cycle of PCR: for example, in amplification of cheA1, a nearly 75% increase in PCR yield over that obtained with solvent alone was obtained (Figure 4). The results seen in what we name the “BSA PCR step” method are consistent with the assumption that BSA denaturation causes the drop in yield-enhancing effect after the fifteenth cycle of PCR. Control experiments where glycerol or distilled sterile water was added at every tenth cycle did not increase the PCR yield, indicating that the effects of BSA are not due to a change in the reaction volume or to BSA effectively acting as a molecular crowder, a property attributed to some of the effects of glycerol in PCR [13]. While the exact mechanisms of the enhancing effect of BSA on PCR yield are not known, the data obtained here and described in the literature [10,12,14,16,18,19] support the hypothesis that BSA may stabilize the DNA polymerase and/or counteract the potential inhibitory effects of high concentrations of organic solvents on DNA polymerase activity. For amplification of cheA4, the DNA fragment with the highest GC content used in this study (73%), an increase in the PCR yield was obtained by adding BSA as well as DMSO to the PCR, but multiple nonspecific amplification products were also detected (Figure 5). To resolve this issue, the BSA PCR step method was next used in combination with a “Touchdown” PCR protocol which has previously been shown to improve PCR specificity [22]. This method produced a single specific band, and nearly doubled the yield that was produced by using the “Touchdown” protocol plus organic solvents alone (96% increase) (Figure 5). These results also suggested to us a way to improve the relatively low efficiency of using whole plasmid site-directed mutagenesis method described in the QuickChange Stratagene Mutagenesis Kit (Stratagene) to introduce mutation in some GC-rich DNA template (here the tlp2 gene, a 2.5 kb fragment of 66% GC). When we used the pUCtlp2 as a template for site-directed mutagenesis, along with mutagenic primers designed to introduce a single base pair substitution within tlp2 (mutagenic primers designed per manufacturer’s website) and the manufacturer’s protocol, the 5,324 kb fragment corresponding to pUCtlp2 was barely visible (Figure 5). Upon completing the mutagenesis protocol per the manufacturer’s instructions, either no colony or a small number of colonies (less than 5 on average) that contained parental plasmids that lacked the desired mutation were obtained. This type of result, characterized by a poor yield of mutagenesis, has previously been recognized as a common pitfall of this method [23,24]. However, when BSA was supplemented to the manufacture’s buffer at every five steps, a clearly amplification product was detected (Figure 5). Upon completion of the manufacturer’s protocol, over 100 colonies with 2/3 carrying the desired mutation (determined by sequencing) were obtained. We also applied the BSA PCR step in an overlap extension protocol [25] to construct a chimeric protein fusion between an A*. brasilense* gene (ATM, 650 bp) and the cyan fluorescent protein (CFP) (720 bp). In the overlap extension PCR, 2 sets of primer pairs are first used to amplify two DNA fragments to be fused that are produced with a short overlapping sequence with one another. A third amplification uses the amplification products of the first reactions as templates, along with the outermost reverse and forward primers to generate chimeric constructs [25]. The amplification of the initial two DNA fragments, ATM and CFP were very efficient using standard PCR protocols and no significant increase could be obtained by using the BSA PCR Step protocol (Data not shown). However, the second, overlap PCR step, produced numerous nonspecific amplification products and little, if any, desired chimeric amplicon (ATM-CFP; Table 1) (Figure 5). The nonspecific amplification products disappeared, when using a “Touchdown” PCR method, but the specific desired chimeric amplicon remained in very low amount, as detected by a very faint 1,370 bp ATM-CFP band (Figure 5). Using the BSA PCR Step method together with a “Touchdown” protocol, resulted in a 72% increase in yield of the ATM-CFP chimeric amplicon (Figure 5). Subsequent cloning and sequencing confirmed that the correct chimeric construct has been obtained.

**Table 1:** Target DNA fragments and primers used in this study

**Target DNA Fragment**	**Primer Name**	**Primer Sequence**
*che1P*	pFUS2Che1p Xho-F	5′ CCCCTCGAGCGCGATGAACTGGTTG
	pFUS2Che1p Eco-R	5′CCCGAATTCATCGGGTTTCATGGGAC
*tlp5P*	Tlp5FUSfor	5′ CCAAGCTTCTGTCCGGCACCGTCTTC
	Tlp5FUSrev	5′ CCCCTGGAGAGGCAGCAGGGTTTCG
*tlp2*	Tlp2-Hind-F2	5′GGAAGCTTGTCACTCGGGCGACTCG
	Tlp2-Xho-R2	5′GGCCTCGAGTCACGCCGAAGGCGGG
*cheA1*	gwCheA1-For2	5′GGGGACAAGTTTGTACAAAAAAGCAG
	CheA1GW-Rev	5′ GGGGACCACTTTGTACAAGAAAGCTG
*cheop1*	CheA1TMup	5′ AACATGTCCCTGCTCAAGCAGCGTTCC
	CheR1dwn	5′ AACATGTCCCTGCTCAAGCAGCGTTCC
*ATM-CFP*	ATM_CFP OL For	5′ GACGTGGAACTGGTGAGCAAGGGC
	ATM_CFP OL REV	5′ CTCGAGTTAAGATCTGTACAG
	Ovl-ATM For	5′ CCGCTCGAGCAGCGCGATGAACTGGTT
	Ovl-ATM Rev	5′ GTGGAACTGGTGAGCAAGGGC
pUC*tlp2*	Tlp2-SDM3-For2	5′ GCGCAAACGACGCCAACGCCAGCA
	Tlp2-SDM3-Rev2	5′ GCGCCTGGCGGTTGCTGGCGTTGG
*cheA4*	CheA4 H47Q-REV	5′ CTTGATGGACTGGACGGCGCG
	CheA4 H47Q-FOR	5′ CGCGCCGTCCAGTCCATCAAG

## Conclusions

While BSA is a powerful co-enhancer of PCR yield when used in combination with DMSO or formamide, its yield-promoting effect seems to act by increasing the range at which the organic solvent used is effective as a PCR additive (formamide or DMSO). BSA used as a co-enhancer with DMSO also increases overall PCR yield. The BSA PCR step protocol evaluated here demonstrate that high yield of traditionally difficult to amplify DNA fragments can be obtained, with a combination of primers of different sequence complementarity to the template they target and of different nucleotide length. The positive effects of BSA, a cost-effective co-enhancer for PCR amplification of GC-rich DNA templates, are not specific to particular DNA sizes or methods as they could be obtained for fragment over 7 kb in length, in overlap extension PCR and site directed mutagenesis applications.

## Methods

### Materials and reagents

PCR reactions were carried out in 2x Go Taq Colorless Master Mix (Promega), 1.00 ng/μl of template DNA. Reactions were also carried out with Failsafe PCR Buffer C and D (Epicentre), in which BSA demonstrated a similar enhancing effect (data not shown). Site directed mutagenesis reactions were carried out in supplied reaction buffer, dNTP mix and *Pfu Turbo* DNA polymerase, according to the manufacturer’s instructions (Stratagene). The genomic DNA of *Azospirillum brasillense* Sp7 was used as the template for all PCR reactions and obtained using the Wizard Genomic DNA Purification Kit (Promega), according to the manufacturer’s recommendations. After extraction, DNA concentration was determined on an Eppendorf Biophotometer and 100 μg/ml were used in each PCR. For amplification of the CFP-encoding gene (overlap extension PCR protocol), the pANT579 vector (a gift from A. Nebenfuehr, University of Tennessee, Knoxville) that carries the CFP gene cloned in a pBSKII (Pharmacia, Biotech) vector derivative was used as a template. The pBBRcheATM template (Bible and Alexandre, upublished) was used to amplify the ATM fragment. Additives used included bovine serum albumin (BSA), dimethylsulfoxide (DMSO), formamide, and glycerol. BSA was obtained from New England Biolabs, DMSO from Acros Organics, and formamide and glycerol were from Fisher Bioreagents (ThermoFischer, Waltham MA). The primers were synthesized and purified by HPLC (Integrated DNA Technologies, IA) and are listed in Table 1.

### Cycling parameters

Polymerase Chain Reactions were carried out in a Mastercycler ep from Eppendorf, in 500 μl thin-walled PCR tubes. The cycling parameters consisted of an initial denaturation step of 95°C for five minutes, followed by a three step cycle comprised of a denaturation step of 95°C for 1 minute, an annealing step of 45 seconds, and an extension step of 72°C for 1 minute per kilobase of target gene, unless otherwise noted this cycle repeated 30 times. This cycling step was followed by a final extension time of 72°C for 10 minutes, and then the reaction was cooled down to 4°C. The reactions that were supplemented with BSA at intermediate steps followed the same cycling parameters as listed above, except that the cycle was manually paused after every tenth steps to allow BSA addition. In control reactions, these same cycling parameters were carried out with the addition of the same volume of sterile distilled water or glycerol added instead of BSA. For the “Touchdown” PCR, the cycling parameters consisted of an initial denaturation step of 95°C for 5 minutes followed by a three step cycle comprised of a denaturation step of 95°C and annealing step for 1 minute in which the temperature was initially set 10°C above the predicted annealing temperature and decreased one degree per cycle, and an extension time of 72°C for 1 minute per kilobase of target gene. Another three step cycle followed this one in which there was an initial denaturation step of 95°C for 1 minute, an annealing step of 45 seconds, and an extension step of 72°C for 1 minute per kilobase of target gene. Unless otherwise noted, the initial cycling step was repeated for fifteen cycles and the second cycling step was repeated for 20 cycles. The final cycling step was followed by a final extension time of 72°C for 10 minutes and was cooled down to 4°C. The reactions that were supplemented with BSA at intermediate steps followed the same cycling parameters as listed above, except that the cycle was manually paused after every five steps to allow BSA addition. The mutagenesis PCR was carried out using the protocol supplied by the manufacturer (Stratagene) with primers Tlp2-SDM3-For2 and Tlp2-SDM3-Rev2 and pUCTlp2 that contains the tlp2 gene cloned in pUC18 (total size of 5324 bp) as a template (Table 1). Cycling conditions for site-directed mutagenesis included an initial 95°C denaturation step, followed by a three-step cycle that included 95°C for thirty seconds, 55°C for 1 minute, and 68°C for 1 min/kb of template. The cycle was repeated 16times. For the overlap extension PCR, CFP was amplified from the plasmid pAN579 and the TM region of cheA was amplified from the plasmid PBBRCheATM. The reaction parameters for each of these was an initial denaturation step of 95°C for five minutes followed by a three step cycle comprised of a denaturation step of 95°C for 1 minute, an annealing step of 45 seconds, and an extension step of 72°C for 1 minute per kilobase of target gene, unless otherwise noted this cycle was carried out 30 times. Following this step, there was a final extension time of 72°C for 10 minutes, and then the reaction was cooled down to 4°C. Results were then viewed on an agarose gel and the bands corresponding to the target genes were cut out and extracted using the QIAquick Gel Extraction Kit (Qiagen). The extracted fragments were then added in a 1:1 ratio to a reaction that was carried out using the same parameters that were used to amplify the individual fragments. This same reaction was carried out following the parameters of the previously detailed BSA PCR step plus Touchdown PCR.

### Data analysis

The resulting PCR products loaded into a 1.0% agarose gel stained with ethidium bromide. The gel was then photographed, and densitometric quantification of amplification products was carried out using the NIS Elements Br 2.30 (Nikon) program as described in [10]. Each PCR was repeated at least 3 times and an average value and standard deviation was recorded from the image analysis. PCR yield increase was calculated by subtracting the yield obtained in control PCR without any additives from the yield obtained in presence of the additive tested. The resulting number was then divided by the yield obtained in PCR without any additives to give the percentage of PCR yield increased due to a certain additive [10].

## Abbreviations

PCR: Polymerase chain reaction; BSA: Bovine serum albumin; DMSO: Dimethyl sulfoxide; CFP: Cyan fluorescent protein.

## Competing interests

The authors declare that they have no competing interest.

## Authors’ contributions

EF designed and carried out all experiments, participated in the interpretation of the results and wrote the first draft of the manuscript. GA participated in the design of the study, interpretation of the results and drafted the manuscript. All authors read and approved the final manuscript.

## References

[B1] Saiki RK Gelfand DH Stoffel S Scharf SJ Higuchi R Horn GT Mullis KB Erlich HA Directed enzymatic amplification of DNA with a thermostable DNA-polymerase Science 1988 239 4839 487 491 10.1126/science.2448875 2448875

[B2] Simpson D Crosby RM Skopek TR A method for specific cloning and sequencing of human hprt cDNA for mutation analysis Biochem Biophys Res Commun 1988 151 1 487 492 10.1016/0006-291X(88)90619-5 3348790

[B3] Dulau L Cheyrou A Aigle M Directed mutagenesis using PCR Nucleic Acids Res 1989 17 7 2873 2873 10.1093/nar/17.7.2873 2654888 PMC317681

[B4] Westwood SA Werrett DJ An evaluation of the polymerase chain-reaction method for forensic applications Forensic Sci Int 1990 45 3 201 215 10.1016/0379-0738(90)90176-Y 2361643

[B5] Mamedov TG Pienaar E Whitney SE TerMaat JR Carvill G Goliath R Subramanian A Viljoen HJ A fundamental study of the PCR amplification of GC-rich DNA templates Comput Biol Chem 2008 32 6 452 457 10.1016/j.compbiolchem.2008.07.021 18760969 PMC2727727

[B6] Li LY Li Q Yu YH Zhong M Yang L Wu QH Qiu YR Luo SQ A primer design strategy for PCR amplification of GC-rich DNA sequences Clin Biochem 2011 44 8–9 692 698 21315705 10.1016/j.clinbiochem.2011.02.001

[B7] Shinoda N Yoshida T Kusama T Takagi M Hayakawa T Onodera T Sugiura K High GC contents of primer 5 ′-end increases reaction efficiency in polymerase chain reaction Nucleosides Nucleotides Nucleic Acids 2009 28 4 324 330 10.1080/15257770902963400 20183585

[B8] Don RH Cox PT Wainwright BJ Baker K Mattick JS Touchdown PCR to circumvent spurious priming during gene amplification Nucleic Acids Res 1991 19 14 4008 4008 10.1093/nar/19.14.4008 1861999 PMC328507

[B9] Shore S Paul N 5 5 Robust PCR amplification of GC-rich targets with Hot Start 7-deaza-dGTP Biotechniques 2010 49 841 843

[B10] Chakrabarti R Schutt CE The enhancement of PCR amplification by low molecular weight amides Nucleic Acids Res 2001 29 11 2377 2381 10.1093/nar/29.11.2377 11376156 PMC55707

[B11] Bachmann B Luke W Hunsmann G Improvement of PCR amplified DNA sequencing with the aid of detergents Nucleic Acids Res 1990 18 5 1309 1309 10.1093/nar/18.5.1309 2320432 PMC330471

[B12] Chakrabarti R Schutt CE 4 4 Novel sulfoxides facilitate GC-rich template amplification Biotechniques 2002 32 866 11962608 10.2144/02324rr04

[B13] Kramer MF Coen DM Bonifacino Juan S Enzymatic amplification of DNA by PCR: standard procedures and optimization Current protocols in cell biology 2001 Appendix 3:Appendix 3F 10.1002/0471143030.cba03fs10 18228288

[B14] Ralser M Querfurth R Warnatz H-J Lehrach H Yaspo M-L Krobitsch S An efficient and economic enhancer mix for PCR Biochem Biophys Res Commun 2006 347 3 747 751 10.1016/j.bbrc.2006.06.151 16842759

[B15] Sarkar G Kapelner S Sommer SS Formamide can dramatically improve the specificity of PCR Nucleic Acids Res 1990 18 24 7465 7465 10.1093/nar/18.24.7465 2259646 PMC332902

[B16] Valetto A Martino DD N, N, N-trimethylglycine (betaine) improves analysis of CDR3 diversification in children reconstituting their immune repertoire after hematopoietic stem-cell transplantation Transplantation 2007 83 7 996 997 10.1097/01.tp.0000258728.02932.02 17460574

[B17] Chang BS Mahoney RR Enzyme thermo stabilization by bovine serum albumin and other proteins - evidence for hydrophobic interactions Biotechnol Appl Biochem 1995 22 203 214 7576258

[B18] Kreader CA Relief of amplification inhibition in PCR with bovine serum albumin or T4 gene 32 protein Appl Environ Microbiol 1996 62 3 1102 1106 8975603 10.1128/aem.62.3.1102-1106.1996 PMC167874

[B19] Woide D Zink A Thalhammer S Technical Note: PCR Analysis of Minimum Target Amount of Ancient DNA Am J Phys Anthropol 2010 142 2 321 327 20229501 10.1002/ajpa.21268

[B20] Tarrand JJ Krieg NR Dobereiner J Taxonomic study of Spirillum lipoferumgroup, with descriptions of a new genus, Azospirillum gen. nov and 2 species, Azospirillum lipoferum (Beijerink) comb nov and Azospirillum brasilense sp. nov Can J Microbiol 1978 24 8 967 980 10.1139/m78-160 356945

[B21] Martin-Didonet CCG Chubatsu LS Souza EM Kleina M Rego FGM Rigo LU Yates MG Pedrosa FO Genome structure of the genus Azospirillum J Bacteriol 2000 182 14 4113 4116 10.1128/JB.182.14.4113-4116.2000 10869094 PMC94601

[B22] Korbie DJ Mattick JS Touchdown PCR for increased specificity and sensitivity in PCR amplification Nat Protoc 2008 3 9 1452 1456 10.1038/nprot.2008.133 18772872

[B23] Liu HT Naismith JH An efficient one-step site-directed deletion, insertion, single and multiple-site plasmid mutagenesis protocol BMC Biotechnol 2008 8 10 10.1186/1472-6750-8-10 19055817 PMC2629768

[B24] Reikofski J Tao BY Polymerase chain-reaction (PCR) techniques for site-directed mutagenesis Biotechnol Adv 1992 10 4 535 547 10.1016/0734-9750(92)91451-J 14543704

[B25] Bryksin AV Matsumura I Overlap extension PCR cloning: a simple and reliable way to create recombinant plasmids Biotechniques 2010 48 6 463 465 10.2144/000113418 20569222 PMC3121328

